# Chinese Milk Vetch Improves Plant Growth, Development and ^15^N Recovery in the Rice-Based Rotation System of South China

**DOI:** 10.1038/s41598-017-03919-y

**Published:** 2017-06-15

**Authors:** Zhijian Xie, Yaqin He, Shuxin Tu, Changxu Xu, Guangrong Liu, Huimin Wang, Weidong Cao, Hui Liu

**Affiliations:** 1 Institute of Soil & Fertilizer and Resources & Environment, National Engineering and Technology Research Center for Red Soil Improvement, Jiangxi Academy of Agricultural Sciences, Nanchang, 330200 P.R. China; 2 College of Resource and Environment, Microelements Research Center, Huazhong Agricultural University, Wuhan, 430070 P.R. China; 3 School of Economics and Management, Key Laboratory of Agricultural Resources and Ecology in Poyang Lake Basin of Jiangxi Province, Jiangxi Agricultural University, Nanchang, 330045 P.R. China; 4 Institute of Agricultural Resources and Regional Planning, CAAS, Beijing, 100081 P.R. China; 5 Jiangxi Monitoring Station of Agricultural Environment, Nanchang, 330001 P.R. China

## Abstract

Chinese milk vetch (CMV) is vital for agriculture and environment in China. A pot experiment combined with ^15^N labeling (including three treatments: control, no fertilizer N and CMV; ^15^N-labeled urea alone, ^15^NU; substituting partial ^15^NU with CMV, ^15^NU-M) was conducted to evaluate the impact of CMV on plant growth, development and ^15^NU recovery in rice-based rotation system. The ^15^NU-M mitigated oxidative damage by increasing antioxidant enzymes activities and chlorophyll content while decreased malondialdehyde content in rice root and shoot, increased the biomass, total N and ^15^N uptake of plant shoots by 8%, 12% and 39% respectively, thus inducing a noticeable increase of annual ^15^N recovery by 77% versus ^15^NU alone. Remarkable increases in soil NH_4_^+^ and populations of bacteria, actinomycetes and azotobacter were obtained in legume-rice rotation system while an adverse result was observed in soil NO_3_^−^ content versus fallow-rice. CMV as green manure significantly increased the fungal population which was decreased with cultivating CMV as cover crop. Therefore, including legume cover crop in rice-based rotation system improves plant growth and development, annual N conservation and recovery probably by altering soil nitrogen forms plus ameliorating soil microbial communities and antioxidant system which alleviates oxidative damages in plants.

## Introduction

The rice growing acreage and production in China are at the forefront of the world, which accounted for 18.7% of global rice growing acreage and 8.7% of global rice production in 2012^1^. The current farmland area is not likely to increase any more, thus improving the yield per hectare and the comprehensive productivity of rice crops are deemed the most effective way to increase the total yield of rice crops^2^. In addition, maintaining the stable and sustainable growth of cereal crops requires foreign substances inputs, such as fertilizers (especially N fertilizer) and agricultural films, and so forth, which have been reported to contribute by more than 50% to the yield of cereals^3^.

The modern intensive system with “high-input, high-output but unfortunately synchronously associated with high resource consumption and environmental costs”^4^, especially applying more mineral fertilizer nitrogen (FN), but associated with less green manure (GM), has lasted over the past three decades. The application rate of FN even has by far exceeded the N demand for targeted yield of crops in some agricultural regions. Although the rice yields dramatically increased in a short term^5^, the N use efficiency remained at a low level (less than 40%) and soil N deposition greatly increased over time^6^, which resulted in remaining nitrate-N in paddy soil profile^7^. This situation has caused a series of problems such as soil acidification and structure damage, water depletion, and altered populations plus activities of soil microbes^8–10^, which in turn negatively influenced the yield-increasing trend of FN year by year^11^. Over-fertilization is not only a waste of resources but also a threat to the soil quality and productivity as well as the food and environmental security such as atmospheric and water pollutions^12, 13^.

In order to meet the demands of food security in the face of an increasing world population and environmental challenge, it is crucial to enhance crop yield and yield stability under non-optimal and adverse growing conditions by a combination of approaches. It is well known that various abiotic stresses (e.g. water logging and drought, etc.) have a profound impact on nutrients uptake of plant tissues and other metabolic activities of plant cells. In plants, exposure to the abiotic stresses will cause oxidative damages to plants by triggering an increase in reactive oxygen species (ROS) production such as hydroxyl free radical (**·**OH-), superoxide radical (O_2_ **·**-) and hydrogen peroxide (H_2_O_2_)^14, 15^, which lead to a disruption in the balance between ROS production and antioxidant quenching activity. Consequently, it results in decrease of nutrients uptake^14^, changes in N metabolism and inhibitions of photosynthesis as well as damages in chloroplast pigments and enzymes in plants^16, 17^, and thus retarding plant growth by disturbing normal metabolic activities of plant cells^18^. The chlorophyll content and malondialdehyde (MDA) are usually used as indicators of the oxidative damage in plants^21^. MDA is a highly reactive three carbon dialdehyde which is produced as a byproduct of fatty acid peroxidation in the cell membrane due to abiotic stresses in plant cells^19^. As to combat the oxidative damages resulted from sorts of adverse conditions, plants have evolved a range of physiological and metabolic responses by activating antioxidant defense systems, which include both the enzymic substances such as catalase (CAT), peroxidases (POD), superoxide dismutases (SOD) and the nonenzymic constituents such as the reduced glutathione (GSH), so as to remove, neutralize and scavenge the ROS and consequently confer tolerance against the environmental stresses^14^.

Cover crops (e.g. Chinese milk vetch, hair vetch, and ryegrass, etc.) grow during periods when the soil might otherwise be fallow after the harvest of main crops. While actively growing, cover crops increase solar energy harvest and C flux into the soil, providing food for soil macro- and micro- organisms^20^. Moreover, cover crops generally have a considerable amount of aboveground and underground biomass^21^. It is important to select cover crops with a high aboveground biomass production to conserve N and estimate the need for supplemental N to meet early growth requirements of succeeding crops, as mineralized N from cover crop biomass would likely not be available until later in the growing season^22^. Also, cover crops possess a strong ability to establish a deep root system to facilitate nutrient uptake from lower soil depths and absorb less available nutrients in the soil profile, thus helping in increasing concentration of plant nutrients in the surface soil layers^23^, and depleting the inorganic N in soil profiles and especially preventing the environmental risks related to NO_3_^−^ leaching, filtration and denitrification in agro-systems^24^. Furthermore, incorporating the cover crop residues as GM into soil not only transfers nutrients (e.g. N, P, and K, etc.) which also are stored in the free seasons for the subsequent crops^25^, but also promotes the decomposition rate of the indigenous soil organic N^26^, both of which are not only advantageous for growth and development of the main crops, but also for the absorption and output of N nutrient.

So far, most of the studies on the cover crops have concentrated on the fate of N in the dry land or growing seasons of main crops^27, 28^. Chinese milk vetch (CMV) as GM in rice or cover crop after the rice harvest shifted lots of microbial genera which functioned in N cycle, and thus increasing the NH_4_^+^ supply and decreasing the soil acidifcation in GM-amended paddy soil^29^. Furthermore, substituting FN with CMV as GM in rice not only improves the N-supplying capacity, the productivity and sustainability of paddy fields, but also mitigates N_2_O emission without compromising rice grain yield of the rice-based cropping system^30, 31^. Nevertheless, few studies are available on the effects of CMV as GM in rice or as cover (or catch) crop after the harvest of rice plants on the physiological traits in rice shoots and roots as well as the annual ^15^N recovery and dynamics of soil biological properties in the winter legume cover crop-rice rotation system. We presumed that CMV as GM incorporates with mineral FN to soil in rice or cover crop after the rice harvest would promote the growth and development of plants as well as the N conservation and recovery in the leguminous CMV-rice cropping system by ameliorating soil microbial communities and antioxidant system in rice plants which can alleviate its oxidative damages in the rice-based rotation system of south China. Hence, the present study was aimed at: 1) illustrating the effect of CMV as GM on the antioxidant system of rice shoot and root, 2) evaluating the annual effect of CMV as GM in rice or cover crop after the rice harvest on the inorganic N contents and microbial communities of top paddy soil in legume winter cover crop-rice rotation system, and 3) understanding the annual recovery of ^15^N-labled fertilizer N in the winter legume winter cover crop-rice rotation system.

## Results

### Biomass and total N uptake in shoot of rice and cover crop plants

Substituting partial FN with CMV in rice was propitious to increase the biomass and total N uptake of shoots in the rice-based cropping system (Table 1). Compared with applying ^15^N-labeled urea as FN alone (^15^NU), substituting partial ^15^N-labeled urea with CMV (^15^NU-M) in rice increased the biomass and total N uptake (N derived from soil, FN and CMV) of plant shoots by 8.2% and 13.4% in rice (~6.6% and 11.2% in straw, ~14.0% and 17.7% in filled grain) and 8.5% and 1.5% in CMV, which led to an increase of 8.2% and 12.0% in the rice based rotation system, respectively.

**Table 1:** Biomass and total N uptake in shoots of rice and CMV plants.

Treatments	Rice				CMV	Rotation system	Rice					Rotation system
	Straw	FG	AG	Shoot			Straw	FG	AG	Shoot	CMV	
	Dry matter weight (×10^3^ kg/ha)						Total nitrogen (kg/ha)					
Control	6.49 c	3.83c	1.26 b	11.6c	0.37c	12.0 c	35.6 c	52.5 c	5.63b	94.4c	11.3b	105.6 c
^15^NU	12.1 b	7.36b	2.54 a	22.0b	1.18b	23.2 b	86.9 b	143.1 b	20.6a	250.6b	39.4a	290.0 b
^15^NU-M	12.9 a	8.39a	2.53 a	23.8a	1.28a	25.1 a	96.6 a	168.5 a	18.8a	284.1a	41.0a	324.7 a
HSD (*P* ≤ 0.05)	0.023	0.001	0.026	0.005	0.023	0.004	0.006	0.008	0.028	0.002	0.046	0.003

^15^NU: ^15^N-labeled urea as FN alone, ^15^NU-M: substituting partial ^15^N-labeled urea with CMV as GM. FG: filled grain, AG: abortive grain. Different letters within the same column indicate significant differences among treatments according to Tukey’s test at *P* ≤ 0.05.

### ^15^N uptake in rice and cover crop shoots

Substituting partial FN with CMV as GM (^15^NU-M) in rice remarkably promoted ^15^N accumulation and *Ndf* in crops of the rice-based rotation system (Table 2). Compared with the treatment of FN alone (^15^NU), substituting partial FN with CMV as GM (^15^NU-M) in rice significantly increased the ^15^N accumulation and *Ndf* by 18.1% and 5.5% in rice straw, by 61.0% and 38.5% in rice filled grain, by 19.1% and 19.5% in CMV plants shoots, while significantly decreased by 21.6% and 10.9% in rice abortive grain, respectively, eventually led to an overall increase in the ^15^N accumulation by 39.5% in the rice-based rotation system.

**Table 2:** The ^15^N accumulation and its proportions to total N in shoots of rice and CMV plants.

Treatments	The ^15^N accumulation in rice and CMV shoots (kg/ha)						*Ndf*(%)			
	Rice				CMV	Rotation system	Rice			CMV
	Straw	FG	AG	Shoot			Straw	FG	AG	
^15^NU	23.8 b	43.1 b	6.06 a	72.5 b	1.31 b	74.4 b	27.3 b	30.1 b	29.4 a	3.29 b
^15^NU-M	28.1 a	69.4 a	4.75 b	102.5 a	1.56 a	103.8 a	28.8 a	41.7 a	26.2 b	3.93 a
HSD (*P* ≤ 0.05)	0.012	0.002	0.008	<0.001	0.034	<0.001	0.037	0.003	0.004	0.016

^15^NU: ^15^N-labeled urea as FN alone, ^15^NU-M: substituting partial ^15^N-labeled urea with CMV as GM. FG: filled grain, AG: abortive grain. Different letters within the same column indicate significant differences among treatments according to Tukey’s test at *P* ≤ 0.05.

### The chlorophyll dynamics (SPAD readings) in leaves at different growing stages of rice plants

**Figure 1:**
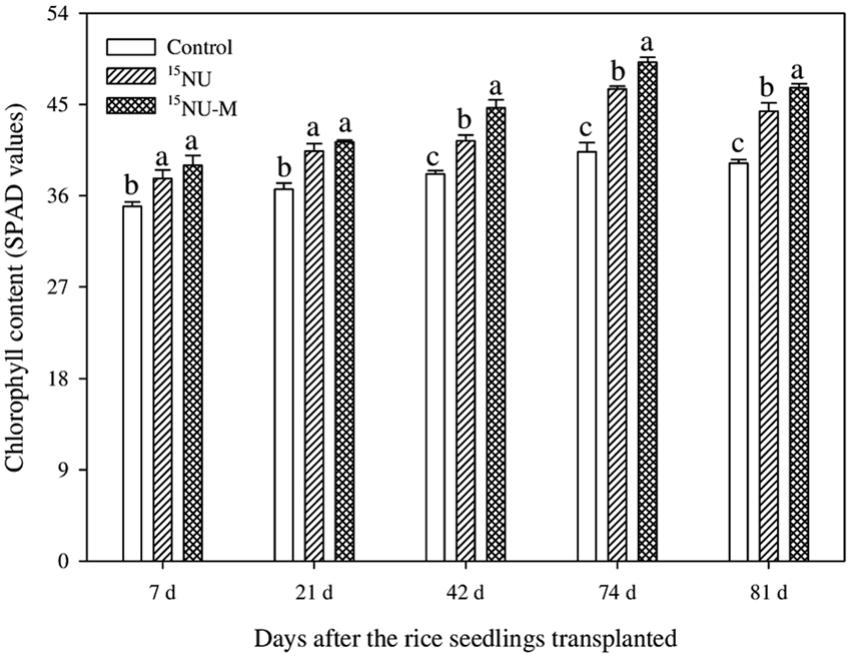
Chlorophyll dynamics (SPAD readings) in leaves during the different growth periods of rice plants. Values are means ± standard error (n = 3). Values followed by different letters in the same pattern bars are significantly different according to Tukey’s test at *P* ≤ 0.05.

Substituting FN with CMV as GM in rice mainly promoted the chlorophyll contents after tillering (Fig. 1). There were no remarkable differences in SPAD readings at 7 and 21 days after transplanting rice seedlings (i.e. the vegetative growth stage alone) between the treatments of ^15^NU and ^15^NU-M. However, the treatment of ^15^NU-M in rice noticeably increased the SPAD readings both at 42 days (i.e. the synchronous stages of vegetative and reproductive growth), 74 and 81 days after transplanting seedlings (i.e. reproductive stage alone) by ~5.2–7.9% versus the treatment of ^15^NU.

### The activities of antioxidant enzymes in rice leaves and roots

**Figure 2:**
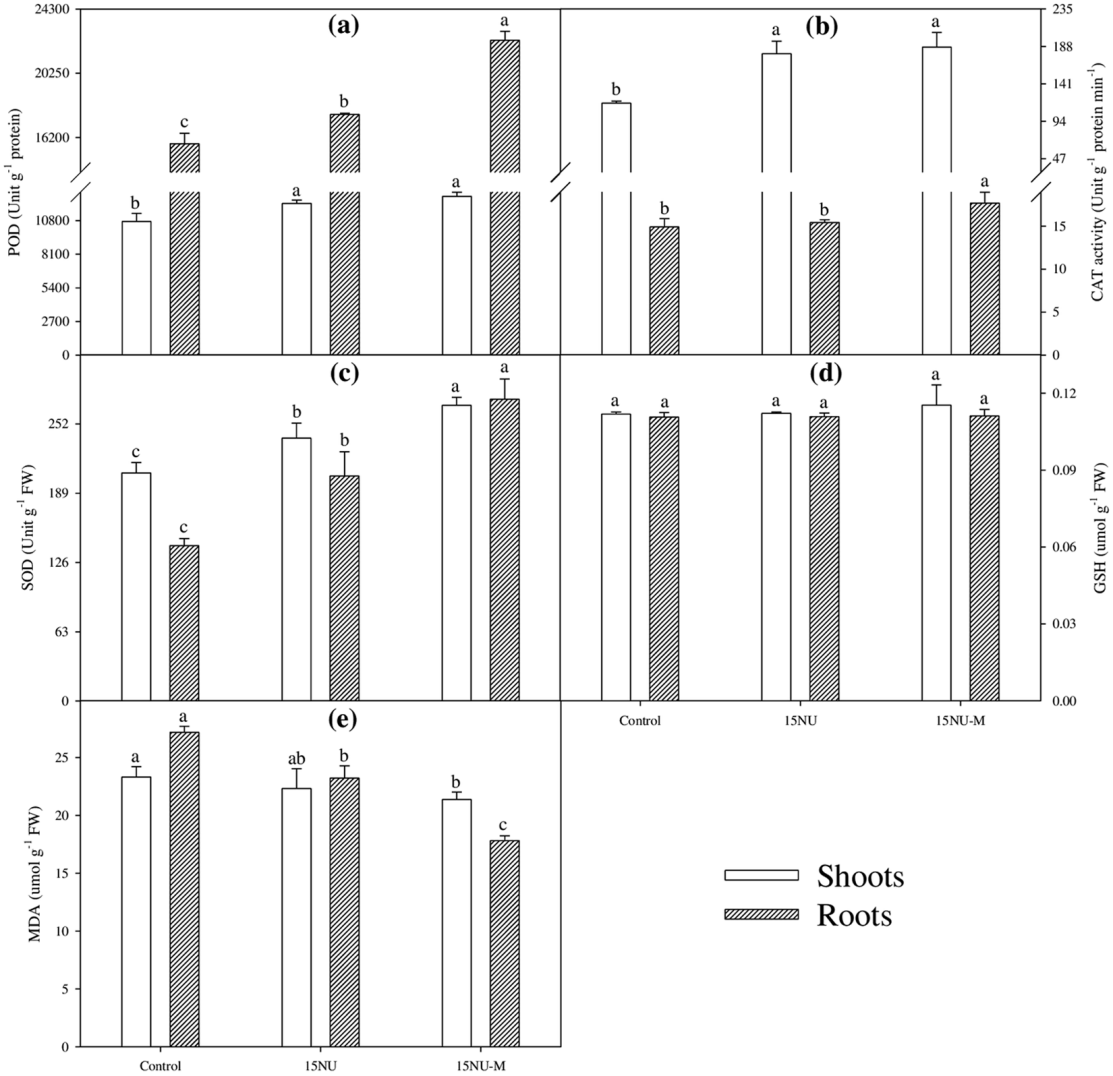
Effects of Chinese milk vetch on the activities of antioxidant enzymes in leaves and roots on 74 days after rice seedlings transplanting. (**a**) peroxidase (POD), (**b**) catalase (CAT), (**c**) superoxide dismutase (SOD), (**d**) reduced glutathione (GSH) and (**e**) malondialdehyde (MDA). FW: fresh weight. Values are means ± standard error (n = 3). Values followed by different letters in the same pattern bars are significantly different according to Tukey’s test at *P* ≤ 0.05.

Fertilization mitigated the oxidative damages and promoted the growth of rice shoots and roots (Fig. 2). Compared with control, the N-treated treatments (^15^NU and ^15^NU-M) remarkably increased the activities of antioxidant enzymes (e.g. SOD, POD, and CAT) by 13.4–60.2% in shoots and 3.5–94.2% in roots, while the MDA content was noticeably decreased i.e. by mean values of 6.3% and 24.5% in shoots and roots, respectively.

Though no remarkable differences were observed in GSH content under condition of the same total rate of N applied to rice plants, the ^15^NU-M pots significantly increased the antioxidant enzymes activities (i.e. SOD, POD, and CAT) by 4.6–12.5% in shoots and 14.6–34.1% in roots, while noticeably decreased the MDA content by 4.3% and 23.3% in shoots and roots respectively, as compared to the treatment of ^15^NU (Fig. 2).

### Annual ^15^N recovery in legume cover crop-rice rotation system

Substituting partial FN with CMV (^15^NU-M) in rice promoted the recovery of ^15^N-labeled fertilizer in legume winter cover crop-rice rotation system (Table 3). When compared with ^15^NU alone, substitution of CMV for partial FN (^15^NU-M) in rice significantly increased the recovery of ^15^N in shoot of rice plants by 77.9% (50.2% and 105.0% in rice straw and filled grains respectively), and by 22.2% in shoot of cover crop plants. Consequently, the ^15^N recovery in the rice-based rotation system was 77.4% higher under the treatment of substituting partial FN with CMV than the treatment of FN alone.

**Table 3:** The ^15^N recovery by shoots of rice and CMV plants in the rice-based rotation system (%).

Treatments	Rice				CMV	Rotation system
	Straw	FG	AG	Shoot		
^15^NU	6.66 b	12.0 b	1.69 a	20.4 b	0.36 b	20.8 b
^15^NU-M	10.0 a	24.6 a	1.70 a	36.3 a	0.44 a	36.9 a
HSD (*P* ≤ 0.05)	0.009	<0.001	0.083	<0.001	0.004	<0.001

^15^NU: ^15^N-labeled urea as FN alone, ^15^NU-M: substituting partial ^15^N-labeled urea with CMV as GM. FG: filled grain, AG: abortive grain. Different letters within the same column indicate significant differences among treatments according to Tukey’s test at *P* ≤ 0.05.

### Inorganic N contents and populations of microbial communities in top paddy soil

Compared with applying FN alone, the ^15^NU-M pots remarkably increased the NH_4_^+^ content by 16.7% as well as the populations of bacteria, fungi, actinomycetes and azotobacter by 36.7%, 14.0%, 18.0% and 21.9% respectively, while noticeably decreased the NO_3_^−^ content by 27.5% at maturity stage of rice plants (Table 4).

**Table 4:** The inorganic N contents and populations of microbes in the top paddy soil of legume-rice rotation system.

Items	NH_4_^+^-N (mg/kg)	NO_3_^−^-N (mg/kg)	Bacteria (CFU 10^8^/g)	Fungi (CFU 10^5^/g)	Actinomycetes (CFU 10^6^/g)	Azotobacter (CFU 10^7^/g)
***Rice growing seasons***						
Control	5.99 c	0.20 c	2.54 c	0.99 c	10.3 c	17.3 c
^15^NU	10.4 b	0.51 a	7.39 b	1.43 b	12.2 b	21.9 b
^15^NU-M	12.1 a	0.37 b	10.1 a	1.63 a	14.4 a	26.7 a
HSD (*P* ≤ 0.05)	0.007	0.018	<0.001	0.021	0.013	0.009
***With cover crop or fallow***						
With cover crop	8.20 a	9.13 b	7.98 a	1.60 b	14.2 a	21.8 a
Fallow	6.23 b	10.8 a	4.67 b	3.05 a	14.9 a	17.6 b
HSD (*P* ≤ 0.05)	0.019	0.031	<0.01	0.008	0.073	0.006

^15^NU: ^15^N-labeled urea as FN alone, ^15^NU-M: substituting partial ^15^N-labeled urea with CMV as GM. Different letters within the same column indicate significant differences among treatments according to Tukey’s test at *P* ≤ 0.05.

Furthermore, growing CMV as winter cover crop after harvesting the rice plants improved the NH_4_^+^ content and the populations of bacteria and azotobacter, whereas decreased the NO_3_^−^ content and population of fungi in paddy soil (Table 4). Comparing with fallow pots after harvesting the rice plants, cultivating CMV as winter cover crop noticeably increased the NH_4_^+^ content by 31.6% as well as the populations of bacteria and azotobacter by 70.9% and 23.9% respectively, while significantly decreased the NO_3_^−^ content and the population of fungi by 15.5% and 47.5% in top paddy soil respectively.

## Discussion

### Shoot Biomass for the rice-based rotation system

Our study showed that substituting partial FN with CMV as GM in rice remarkably increased the shoots biomass (especially the filled grain and straw of rice) versus FN alone (Table 1). Previous studies indicated that incorporating crop residues (e.g. rice straw, CMV stubbles, etc.) dramatically increased the number of spikelets per panicle and the weight of 1000-grain as well as the grains per panicle and panicle number per unit area^32, 33^.

The balanced-supply of nutrients in paddy soil is important for improving plant growth and increasing yields or biomass^34^. Obviously, in addition to the primary macro-nutrients (N, P, and K), CMV plants also contains sorts of medium- and micro-nutrients (e.g. Ca, Mg, Si, and Zn, etc.), which promote and balance the sustainable nutrients supply after incorporating its stubble as GM to paddy soil^35, 36^. Efthimiadou *et al*.^37^ further proved that combining FN with organic manure enhanced the photosynthetic rate and stomatal conductance of rice plants, and led to promoting the dry matter accumulation plus stabilizing the rice yield versus FN alone.

Winter legumes within rice systems played an important role in sustaining plant production through non-N benefits, such as reducing weed population and the incidence of root and leaf diseases in subsequent crops^38, 39^, increasing P and K availability in plough layer of paddy soil which were propitious to promote the N absorption of rice plants and increase the plumpness of rice grains respectively^40^.

### Nitrogen uptake and ^15^N recovery for the rice-based rotation system

Substituting partial FN with CMV as GM in rice dramatically increased the N or ^15^N absorption and recovery by plants from the rice based cropping system (Tables 1 and 2). The plant root system plays a decisive role in N uptake from soil and the better soil environments would promote root growth (e.g. root density, biomass, activity, and nutrients uptake capacity, etc.)^41, 42^. It has been confirmed that the spatial distribution, continuity and stability of soil porosity are key factors that directly affect the root growth and nutrients transport^43^. Compared with applying FN alone, combining FN with organic manures not only improved the total porosity as well as agglomeration degree and stability of aggregates in paddy soil^44^, but also promoted mineralization potential and mineralization rate of soil organic N and enhanced the ability of resistance and resilience of soil microbes against heat stress^45, 46^, thereby improving N-supplying capacity and availability^47^, and accordingly promoted N absorption and accumulation in plants.

Mineral FN applied to the rice-based cropping field goes either to the plant by absorption (plant recovery), or remains in the soil, or releases and lost to the environment. Our study showed that substituting partial ^15^N-labeled FN with CMV in rice significantly increased the absorption and recovery of ^15^N over the ^15^N-labeled FN alone under the same rate of total N (Tables 2 and 3). It is well known that low N rate increases N use efficiency. In this study, replacing part (~20% of total) of the mineral FN by CMV as GM consequently reduced the quantity of fertilizer-N applied to the crop, thus essentially increasing the ^15^N recovery.

Furthermore, utilizing legume winter crops efficiently reduced N losses through NH_3_ volatilization, N_2_O emission and NO_3_^−^ filtration from rice-based rotation system and kept more ^15^N-labeled urea in soil after the rice harvest^48^, which could be attributed to the fact of incorporating CMV as GM increased the NH_4_^+^ content while decreased the NO_3_^−^ content in paddy soil^30^, thus resulting in more ^15^N retention in microbial biomass or adsorption on the surface of soil particles in paddy field^49^. Growing winter crops as cover crop after the main crop harvest can not only intercept or absorb the residual ^15^N in soil profile through its developed root network, but may also extract and uptake residual ^15^N from deep soil profile to top soil by the downward growth of root^50^, thereby further improving the annual ^15^N recovery in the winter leguminous CMV-rice cropping system versus the fallow-rice cropping system (Table 3).

### Chlorophyll content and activities of antioxidant enzymes of rice plants

The continuous flooding conditions of paddy soil not only induced the deficiency of oxygen accompanied with accumulation of hazard substances (e.g. CO_2_, Mn, and Fe^2+^, etc.) as a result of decreasing the availability of oxygen to roots^15, 51^, but also increased the rate of ROS (e.g. O_2_ **·**-, H_2_O_2_ and **·**OH-, etc.) production which directly attack membrane lipids and inactivate SH- containing enzymes^52^. Consequently, deleterious results in the form of premature senescence and leaf chlorosis, growth cessation and yield losses have been observed^15^. Our study indicated that substituting partial FN with CMV as GM in rice significantly increased the chlorophyll content (SPAD readings) as well as the activities of SOD, POD and CAT, while remarkably decreased the MDA content in rice root as compared to applying fertilizer N alone (Figs 1 and 2), which revealed that substituting CMV as GM for partial FN in rice promoted plant growth mainly due to its positive role in mitigation of oxidative damages *in vivo* of rice roots. Therefore, it might further logically presume that incorporating FN with CMV as GM improved the growth and development of rice plants by alleviating the adverse impacts of continuous waterlogged conditions of paddy soil on roots and shoots, which in turn might have induced an increase in nutrients absorption (especially the N nutrient), transition and accumulation as well as its metabolism plus the promotion of photosynthesis, and consequently resulted in increasing the biomasses or yields of rice plants.

### Inorganic N content and microbial communities in paddy soil for the rice-based rotation system

Our study showed that substituting partial FN with CMV as GM in rice significantly increased the NH_4_^+^ content and the populations of soil microbes (i.e. bacteria, fungi, actinomycetes and azotobacter), but decreased the NO_3_^−^ content in paddy field versus applying FN alone (Table 4). Our previous study indicated that substituting CMV for partial FN in rice significantly decreased the nitrate reductase (*NaR*) activity^31^, which probably inhibited the process of oxidizing the NH_4_^+^ to NO_3_^−^ in paddy soil. Incorporating CMV into paddy soil improved total N contents, the quantity and quality of soil organic C fractions^47, 53, 54^, which were the major substrates for microbial growth.

In addition, this study further indicated that planting legume CMV as cover crop after the rice harvest dramatically increased the NH_4_^+^ content and the populations of bacteria and azotobacter in paddy soil (Table 4). The increased rhizodeposition (organic compounds released by roots during plant growth) was considered as one of the main factors affecting microbial communities^55^. Cultivating cover crops without N fertilization combined with no-till conservation practice was beneficial for increasing ground soil cover in winter and spring^56^, modifying soil temperature and increasing water holding capacity^57^, which induced higher immobilization of NH_4_^+^ and microbial functional activity and diversity versus bare fallow treatment^49, 58, 59^.

In addition, remarkable decrease in NO_3_^−^ content and population of fungi in paddy soil were observed in the pots with cover crop when compared with the fallow pots (Table 4). Such trends could be better explained in terms of the assumption that the cultivation of legume cover crops led to notably lower diversity of fungal populations, especially the abundance of fungi genera *Mortierella* and *Cryptococcus*, which were positively correlated with soil NO_3_^−^-N^60^. Therefore, the practice of including legume cover crops in rotation systems (e.g. rice-based rotation system) in arable land will permit more sustainable production not only through increasing nutrients (e.g. nitrogen, etc.) in rhizosphere soil, but also via breaking of pathogen lifecycles. However, Detheridge *et al*.^60^ also indicated that cultivating legume cover crops resulted in a negative correlation of soil NO_3_^−^ content with populations of AMF (arbuscular mycorrhizal fungi) and other root-associated fungal groupings. Hence, more field experiments should be carried out to further evaluate the effects of different genotypes of cover crops on the functional diversities and populations or abundances of soil microbes (e.g. fungi and bacteria, etc.) in different types of arable soils, rotations and tilling systems.

## Materials and Methods

### Experimental site, soil, climate and crops

**Figure 3:**
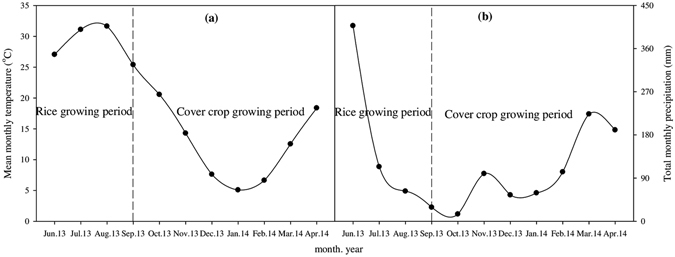
Mean monthly temperature (**a**) and total monthly precipitation (**b**) at the experimental site.

A pot experiment was conducted in greenhouse (28°33′44.47′′ N; 115°56′15.09′′ E; 26.0 m masl) with ventilation equipments from May 2013 to April 2014. The experimental site has a subtropical monsoon climate, characterized by heavy rain from April to June and seasonal drought from September to December. The average annual temperature is 17.8 °C and average annual rainfall is 1546 mm. The annual sunshine is 1603 h. The average frost-free period lasts for 276 d. The average monthly temperature (°C) and total monthly precipitation (mm) at the experimental site from June 2013 to April 2014 are shown in Fig. 3.

The paddy soil derived from Quaternary red soil was obtained from Fengcheng Jiangxi, China (28°07′ N; 115°56′ E; 25.4 m masl). Soil samples were air-dried at room temperature for 2 weeks, and passed through a 2 mm sieve prior to laboratory analysis. Soil pH (soil: water ratio was 1: 2.5), total N (Kjeldahl method), extractable P (the Olsen′s method) and available K (with ammonium acetate) were determined following procedures described by Page *et al*.^61^. The organic C content was determined according to the method described by Walkley & Black^62^. The soil bulk density (SBD) was measured by taking soil cores with a predefined steel cylinder from the undisturbed top soil, oven-dried at 105 °C for 24 h and weighed, then the SBD was calculated as the ratio of dry soil weight (g) to the volume of soil contained in a predefined steel cylinder^63^. The main properties of top soil are shown in Table 5.

**Table 5:** The agro-characteristics of top paddy soil prior to the experiment.

pH	SOC (g/kg)	TN (g/kg)	TP (g/kg)	TK(g/kg)	Available N (mg/kg)	NH_4_^+^ (mg/kg)	NO_3_^−^ (mg/kg)	Olsen-P (mg/kg)	Available K (mg/kg)	SBD (g/cm^3^)	PSD (%)		
											Sand	Silt	Clay
5.41	16.0	1.35	0.43	31.7	102.5	18.7	1.15	8.52	68.0	1.47	14.3	60.7	25.1

SOC: soil organic carbon, TN: total N, TP: total P, TK: total K, SBD: soil bulk density, PSD: particle size distribution.

The legume winter cover crop used in this experiment was Chinese milk vetch (CMV, *Astragalus sinicus* L.), which is quite popular in rice producing areas of south China, and was provided by the germplasm nursery of JAAS (Jiangxi Academy of Agricultural Sciences), China. The rice (*Oryza sativa* L., cv. Qianyou-1) seeds were provided by Zhejiang Nongke Seeds Industry Co. Ltd., China.

### Experimental design

The experiment included three treatments arranged in a randomized complete design with three replications each: (1) control (no fertilizer N and CMV), (2) applying ^15^N-labeled urea as FN alone (^15^NU), (3) substituting partial ^15^N-labeled urea with CMV as GM (^15^NU-M).

The CMV plants were grown in the field outside the experimental area. The fresh vetch, which contained 41.8 g C kg^−1^, 3.35 g N kg^−1^, 0.22 g P kg^−1^, 2.75 g K kg^−1^, a C-to-N ratio of 12.5 and was having a moisture content of 91.6%, was harvested at the full-bloom stage and chopped to about 5 cm length. The chopped CMV was incorporated into pots at its equivalent N rates based on dry matter and N concentration.

### Cultivating rice plants in summer seasons

Each waterproof stainless steel pot (40 cm length, 40 cm width and 30 cm height) contained 47 kg of the air-dried soil (prepared as described above). The applying rate of fertilizers to monocropped rice was: 0.12 g N kg^−1^ soil^−1^ (urea), 0.05 g P kg^−1^ soil^−1^ (single superphosphate) and 0.10 g K kg^−1^ soil^−1^ (potassium chloride). Mineral N, P and K fertilizers were applied as basal fertilizer one day before transplanting the rice seedlings. The fresh vetch straw as GM was applied at the rate of 7.7 g kg^−1^ soil^−1^ (equaling to ~20% of FN) in ^15^NM treatment for the following monocropped rice season, 30 days prior to rice seedlings transplanting, mechanically fully mixed within paddy soil, and then flooded up to 3~5 cm depth. All of the N-fertilized treatments were fertilized with the same total rate of N nutrient which was either from FN alone or both from FN and GM. The ^15^N-labeled FN was urea and with an abundance of 10 atom% (provided by Shanghai Research Institute of Chemical Industry).

The 25-days-old rice seedlings were transplanted into the prepared stainless steel containers (described above) with six caves and two rice seedlings per cave for each container during the last-ten days of May 2013. During the growing period of rice plants, the soil water layer was maintained at 3~5 cm depth by monitoring every day unless the soil was naturally dried 20 days prior to the harvest of rice plants. At physiological maturity, all of rice plants were harvested during the last-ten days of September and grains were artificially separated from straw of rice plants in the whole pot.

### Cultivating winter leguminous cover crop

After the rice harvest, each treatment (as described above) was split into two sub-treatments. One of these were planted with winter cover crop (C) and the other part was kept fallow (F) during the late September 2013 to late April 2014. The cover crop was CMV and seeded at a rate of 30 kg ha^−1^ on the non-tilled paddy soil with the residual soil moisture. No fertilizer was applied during its growing periods to observe the fate of residual effect of ^15^N on cover crop. Weeds were allowed to grow in the weedy fallow pots but were removed from the other pots by manual weeding. The above ground fresh vetch was harvested at the full-bloom stage in the whole pot, and then measured the total N content of vetch plant as well as its ^15^N abundance.

### Sampling and chemical analysis

Soil samples (0~20 cm) from all pots were collected regularly for an analysis of soil mineral N (NH_4_^+^ and NO_3_^−^) content at the maturity stage of rice and full-bloom stage of CMV plants with a soil corer (∅ 1.5 cm) respectively. At each sampling pot, five soil cores (0~20 cm) were randomly collected and then mixed thoroughly inside a plastic bucket to form individual bulked pot soil samples. After removing visible roots and stones, the soil samples were divided into two parts. One of them was sieved through <0.149 mm, then labeled and stored in plastic bags for immediately testing the soil NH_4_^+^ and NO_3_^−^ contents, and the remaining samples were frozen at −20 °C prior to analyze the soil microbes.

Soil mineral N (i.e. NH_4_^+^ and NO_3_^−^) was extracted with 2 M KCl at a 5:1 ratio (KCl: soil, v/v)^64^, and the filtrates were stored at 4 °C for less than 1 day before analysis. The NH_4_^+^ and NO_3_^−^ contents of filtrates were analyzed with a discrete auto analyzer (SmartChem TM200, USA)^65^. Total N in soil was determined by sulfuric acid digestion and Kjeldahl distillation^66^.

The main microbes in soil samples, such as bacteria, actinomycetes, fungi, and azotobacter were counted with traditional dilution plate counting method^67^. In brief, the mixture of fresh soil (~10 g) and sterilized water (~90 ml) was vigorously shaken on a swirl mixer, and then settled for 10 min. The overlying suspension containing microbes was further diluted with the sterilized water at a rate of 1:10. The suspension was plated out on the beef-protein medium, Gauserime synthetic medium, potato dextrose agar medium and Ashby medium for bacteria, actinomycetes, fungi and azotobacter respectively. Then the plates were incubated at (28 ± 1) °C. The formation of colonies was observed regularly every day and the enumeration was performed after incubation for 2~3 d for the bacteria, 10 d for the actinomycetes, 3~5 d for the fungi and 3~4 d for the azotobacter. Colony-forming unit (CFU) per gram of wet soil was calculated for each sample, but the final results were expressed as CFU per gram dry weight (CFU·g^−1^·dw^−1^) based on the water coefficient of the fresh soil samples.

Two rice plants in each pot for biological activity assays were sampled 74 days after transplanting seedlings and separated into two parts (shoots and roots) which were stored in a refrigerator at −80 °C. The rest of rice plants in each pot were harvested at maturity stage and separated into three parts (straw, filled grains and abortive grains) and the CMV plants were sampled at full-bloom stage. Both types of plant samples i.e. rice and fresh vetch were weighed after de-enzyming (105 °C, 30 min) and oven-drying (70 °C, 24 h). All of the dried plant samples were ground and sieved to pass through a 0.25-mm sieve before laboratory analysis. Total N in plant samples was determined by sulfuric acid digestion and Kjeldahl distillation.

### Determination of ^15^N abundances

To determine ^15^N abundance, 0.500 g plant samples were digested in H_2_SO_4_–H_2_O_2_ in the glass vials and stream distilled with 10 ml 40% (w/v) NaOH in the flask. The distillates were collected with 25 ml 2% (w/v) H_3_BO_3_ and acidified with few drops of 0.05 mol·L^−1^ H_2_SO_4_, then dried in an oven at 60 °C. The ^15^N abundance of plants was determined by the method proposed by Buresh *et al*.^68^ and the isotope mass spectrometer was Thermo-Fisher Delta V Advantage IRMS, USA.

### Determination of chlorophyll and malondialdehyde (MDA) in rice plants

Leaf chlorophyll contents were non-destructively measured by *in vivo* procedure using a portable Soil Plant Analysis Diagnostic Meter (SPAD-502, Minolta Camera Co., Osaka, Japan) during the critical growing period of rice plants. Five youngest fully expanded functional leaves of each plant were selected for recording the SPAD readings at 7, 21, 42, 74 and 81 days after the rice seedlings were transplanted. Six plants were randomly selected in each pot, and 3 SPAD readings per each leaf, including one reading around the midpoint of leaf blade and 2 readings at 3 cm apart from the midpoint were determined and then averaged as the mean SPAD reading of the leaf^69^. Then, average SPAD readings were calculated for each treatment.

The MDA content is usually used to indicate the oxidative damage to lipids according to Shah *et al*.^14^ using thio-barbituric acid (TBA). The absorbance of the MDA was read at 532 nm and the blank was 0.25% TBA in 10% trichloroacetic acid (TCA). The concentration of lipid peroxides together with oxidatively modified proteins of shoot or root tissue were quantified in terms of MDA content using an extinction coefficient of 155 mM^−1^ cm^−1^ and expressed as µmol g^−1^ fresh weight (FW).

### Measurements of the activities of antioxidant enzymes in rice shoot and root

For determination of the antioxidant enzymes activities in shoot and root of rice plants, protein extract was prepared according to the method of the Chang & Koa^70^ with some modifications. In brief, washed fresh shoots or roots (0.5 g) were ground in liquid N_2_ and then extracted with extraction buffer containing 0.05 M Tris-HCl (pH 7.0), 3 mM MgCl_2_ and 1 mM EDTA. The homogenate was centrifuged at 12,000 rpm for 15 min at 4 °C, and the supernatant was collected in another falcon tube for the determination of enzymes activities.

The activities of catalase (CAT) and superoxide dismutase (SOD) were assayed according to the method described by Shah *et al*.^14^. The CAT assay mixture in a total volume of 1.5 ml contained l000 µl of 100 mM KH_2_PO_4_ buffer (pH 7.0), 400 µl of 200 mM H_2_O_2_ and 100 µl enzyme. Decrease in H_2_O_2_ was monitored at 240 nm (extinction coefficient of 0.036 mM^1^ cm^−1^) and expressed as unit g^−1^ protein min^−1^. The SOD assay mixture in a total volume of 3 ml contained 50 mM sodium carbonate-bicarbonate buffer (pH 9.8), containing 0.1 mM EDTA, 0.6 mM epinephrine and enzyme. Epinephrine was the last component to be added. The adrenochrome formation during the next 5 min was recorded spectrophotometrically at 470 nm and is expressed as unit g^−1^ protein FW. One unit of SOD activity is defined as the amount of enzyme required to cause 50% inhibition of epinephrine oxidation under the experimental conditions. The activity of peroxidase (POD) was also spectrophotometrically assayed^14^. The increase in absorbance was measured at 470 nm (extinction coefficient 26.6 mM^−1^ cm^−1^) and is expressed as unit g^−1^ protein min^−1^.

The reduced glutathione (GSH) content can be estimated from the difference between total glutathione and GSSG and expressed as µmol g^−1^ FW. Briefly, the total glutathione, namely GSSG (oxidized) and GSH (reduced) were spectrophotometrically determined according to the methods described by Griffith^71^ and Hodges *et al*.^72^. Total glutathione was measured in a reaction mixture containing 400 µl solution A, 320 µl solution B, 400 µl 1:25 dilution of supernatant in 0.5 M KH_2_PO_4_ (pH 7.0), and 80 µl of 3.0 mM NAPDH. The reaction rate to estimate total glutathione was measured by following the change in absorbance at 412 nm for 5 min. For GSSG estimation similar procedure was followed except that 1.0 ml of 1:10 diluted supernatant in 0.5 M KH_2_PO_4_ (pH 6.5) was first incubated with 20 µl 2-vinylpyridine at 25 °C for 1 h to derive GSH. GSH and GSSG standards were between 0 and 18 µM in 5% (w/v) 5-sulphosalicylic acid diluted appropriately with 0.5 M KH_2_PO_4_ (pH 7.0).

### Statistical analysis

Proportions of ^15^N derived from FN (*Ndf*) was calculated by using Eq. 1 ^73^:

1 $$Ndf( \% )=\frac{a-b}{c-b}\times 100$$

where *a* is the atom % ^15^N abundance in plant samples, *b* is the natural atom % ^15^N abundance (0.365%), *c* is the atom % ^15^N abundance of FN.

^15^N amount absorbed by plant was calculated according to Eq. 2:

2 $${}^{15}N(g)=W\times N \% \times Ndf\, \% $$

where *W* is the plant sample weight (g), *N*% is the N concentration in plant samples, *Ndf* % is the N proportion derived from FN.

Nitrogen recovery (*NR*) of plant was calculated using Eq. 3 ^74^.

3 $$NR( \% )=\frac{{}^{15}N(g)}{R(g)}\times 100$$

where *R*(*g*) is the amount of FN applied in each pot (g N/pot).

Values obtained from different treatments were subjected to ANOVA tests. Separation of means was performed on significant ANOVA tests by Tukey HSD (*P* ≤ 0.05) using the SAS (v.9.1) package (SAS Institute, 2009).

## Conclusions

Substituting partial FN with CMV as GM in rice significantly promoted the rice plant growth and development mainly due to an increase in the antioxidant enzymes activities and chlorophyll contents and decrease in the oxidative damages (e.g. MDA content) in plant tissues (especially the roots). These in turn led to an increase in biomass accumulation, uptake of total N and ^15^N uptake in shoots of rice (especially in filled grain) and cover crop, and thus resulting in noticeable increase of ^15^N recovery versus the treatment of FN alone in the legume winter cover crop-rice rotation system under the same total N rate applied to rice plants. In addition, legume-rice rotation system noticeably increased NH_4_^+^ content and populations of soil microbes (e.g. bacteria, actinomycetes and azotobacter) in paddy soil versus the fallow-rice rotation system, however, an adverse result was obtained in soil NO_3_^−^ content. Therefore, it can be concluded that including legume winter cover crops in rice-based rotation system considerably promoted crop growth, development and production especially that of the economical yields (e.g. rice, wheat, and maize, etc.). This enhanced production consequently improved N conservation and recovery which probably resulted from the significant alteration of soil N forms plus the amelioration of soil microbial communities and antioxidant system which can alleviate the oxidative damages in plants.

However, it is imperative to conduct long-term field experiments to further evaluate the impacts of different genotypes of cover crops on the temporal and spatial variances of antioxidant system dynamics in crops as well as the functional diversities and populations of microbial communities in the rhizosphere soil and the N recoveries in various rotation systems. Moreover, the unrevealed potential impacts of legume GMs or cover crops after the rice harvest on the organic N pool of paddy soil also are worth further investigations in the rice-based rotation ecosystem.
